# Electronic Characteristics, Stability and Water Oxidation Selectivity of High-Index BiVO_4_ Facets for Photocatalytic Application: A First Principle Study

**DOI:** 10.3390/nano13132023

**Published:** 2023-07-07

**Authors:** Zhiyuan Zhang, Yuqi Xiang, Zhihong Zhu

**Affiliations:** 1 College of Advanced Interdisciplinary Studies & Hunan Provincial Key Laboratory of Novel Nano Optoelectronic Information Materials and Devices, National University of Defense Technology, 410073 Changsha, China; 2 Nanhu Laser Laboratory, National University of Defense Technology, Changsha 410073, China

**Keywords:** density functional theory, high-index facets, photocatalysis, oxygen evolution reaction

## Abstract

Some high-index facets of BiVO_4_, such as (012), (210), (115), (511), (121), (132) and (231), exhibit much better photocatalytic performance than conventional (010) and (110) surfaces for water splitting. However, the detailed mechanisms and stability of improved photocatalytic performance for these high-index BiVO_4_ surfaces are still not clear, which is important for designing photocatalysts with high efficiency. Here, based on first principle calculation, we carried out a systematic theoretical research on BiVO_4_ with different surfaces, especially high-index facets. The results show that all of the high-index facets in our calculated systems show an n-type behavior, and the band edge positions indicate that all of the high-index facets have enough ability to produce O_2_ without external bias. Electronic structures, band alignments and formation enthalpy indicate that (012), (115) and (132) could be equivalent to (210), (511) and (231), respectively, in the calculation. Oxidation and reduction potential show that only (132)/(231) is stable without strongly oxidative conditions, and the Gibbs free energy indicates that (012)/(210), (115)/(511), (121) and (132)/(231) have lower overpotential than (010) and (110). Our calculation is able to unveil insights into the effects of the surface, including electronic structures, overpotential and stability during the reaction process.

## 1. Introduction

Water splitting based on photoelectrochemical (PEC) catalysis has received increasing attention in recent years, as it is one of the most viable methods to solve the energy crisis without any environment pollution [1,2,3]. Great efforts have been concentrated on designing and fabricating suitable and sustainable photocatalysts in the past few years [4,5,6,7,8]. For the water splitting reaction, a lot of electron–hole pairs can be generated under sunlight, and these electron–hole pairs can separate and transfer to the surface of photocatalysts. Then, H^+^ would be reduced by electrons and produce H_2_, while in contrast, H_2_O would be oxidized by holes and produce O_2_. Therefore, efficient charge transfer and long carrier lifetimes are necessary to ensure the excellent photocatalytic performance [9,10,11]. Furthermore, the conduction band minimum (CBM) and the valence band maximum (VBM) should be above the reduction potential of H^+^/H_2_ and below the oxidation potential of O_2_/H_2_O, respectively [12,13,14]. In this condition, the photogenerated electrons have the ability to reduce proton and the holes have the ability to oxidize H_2_O.

BiVO_4_ is one of the most studied materials for photocatalytic applications due to its outstanding photocatalytic properties [15,16,17,18,19]. In order to design even better performing systems, an in-depth comprehension of improvement strategies, such as crystal facets engineering [20,21,22,23,24,25], is still required. The conventional BiVO_4_ is the nanocrystal with a corner-cut truncated bipyramidal morphology [26,27,28]. Recently, BiVO_4_ widely covered with high-index facets was fabricated and showed an improved oxygen evolution reaction (OER) performance. For instance, the OER performance on (132), (231) and (121) high-index facets was three to five times higher than their (010) and (110) low-index counterparts [29]. BiVO_4_ bounded by multiple high-index (012), (210), (115) and (511) exhibits much higher photocatalytic O_2_ evolution performance (more than two orders of magnitude) compared with conventional BiVO_4_ material [30]. However, there is still a lack of detailed research about the high-index facets, especially the mechanisms of improved photocatalytic performance for high-index facets and the stability of different crystal facets, which has great significance for further improving the photocatalytic performance and promoting the practical application of photocatalysis. Therefore, it is necessary to investigate the mechanisms of the improved photocatalytic performance and stability of these high-index facets.

In this investigation, we report a comprehensive theoretical analysis on BiVO_4_ (012), (210), (115), (511), (121), (132) and (231) high-index facets according to density functional theory (DFT) calculations. We focus on the electronic structure, band edge position, standard formation enthalpy and Gibbs free energy of BiVO_4_ with different facets. The electronic structure of BiVO_4_ is investigated by calculating the partial density of states (DOS). The band edge positions are the focus of analyzing the photocatalytic mechanism. The stability is investigated using thermodynamic reduction potential and oxidation potential, and the photocatalytic activities of BiVO_4_ with different facets are studied via analysis of the Gibbs free energy.

## 2. Materials and Methods

All of the theoretical calculations were based on DFT, as implemented in the Vienna ab initio simulation package (VASP) [31]. The projector-augmented wave (PAW) method was adopted and the generalized gradient approximation (GGA) functional of Perdew, Burke and Ernzerhof (PBE) was selected to describe the interaction between electrons [32,33]. Hubbard U-corrections to the d electrons of V (U_3d_ = 2.7 eV) was performed to correct the self-interaction error during the electronic structure calculations, which has been proven to provide a suitable value [34]. The cut-off kinetic energy was 400 eV for plane wave functions. The convergence criterion for energy was 10^−5^ eV and 10^−7^ eV for optimization and zero-point energy (ZPE) calculation, respectively, and the convergence criterion residual force was set to 0.01 eV/Å. The Monkhorst–Pack k-point grids were set to 7 × 7 × 5 for unit cells and 5 × 5 × 1 for surface systems with Brillouin zones, except for ZPE calculation. For ZPE, only the gamma-point was chosen due to this calculation being the correction for individual OH, O and OOH radicals on the BiVO_4_. In order to avoid the interactions between layers, a vacuum layer more than 20 Å in thickness was placed above the surface systems. Moreover, the computational hydrogen electrode (CHE) model was adopted for OER calculation [35], and an implicit solvent model was used to correct the free energy in this process, as implemented in VASPsol [36]. The solvent is set to water.

## 3. Results and Discussion

### 3.1. Geometric Optimization

**Figure 1:**
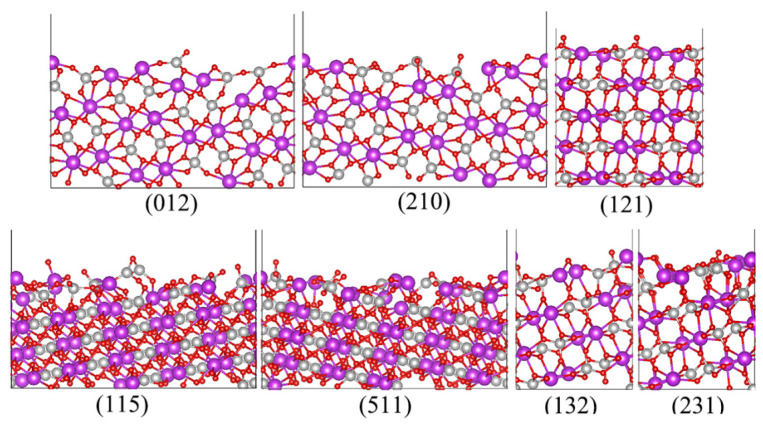
Optimized geometric structures for BiVO_4_ with different facets. The purple, silver and red spheres represent Bi, V and O, respectively.

In our calculations, the unit cell of BiVO_4_ was optimized at first. Generally speaking, the photocatalyst BiVO_4_ has two crystalline phases, named monoclinic scheelite (m-) and tetragonal scheelite (t-). Assuming that *c* is the longest axis, the space group of t-BiVO_4_ is I4_1_/a with the lattice parameters *a* = *b* = 5.15 Å, *c* = 11.72 Å, *α* = *β* = *γ* = 90°, and the m-BiVO_4_ is I2/b (*a* = 5.19 Å, *b* = 5.09 Å, *c* = 11.70 Å, *α* = *β* = 90°, *γ* = 90.4°) or C2/c (*a* = 7.27 Å, *b* = 11.70 Å, *c* = 5.09 Å, *α* = *γ* = 90°, *β* = 135°) as it employs different methods to select the unit cell, and the unit cell of m-BiVO_4_ can spontaneously transform into t-BiVO_4_ if the optimization of the BiVO_4_ unit cell is fully relaxed [37]. Therefore, the unit cell of BiVO_4_ after optimization may be t-BiVO_4_. Due to the m-BiVO_4_ and t-BiVO_4_ having a similar geometric structure, electronic structure, surface energy and work function, all of the calculated results obtained for t-BiVO_4_ can probably be extrapolated for m-BiVO_4_ [38]. In our calculation, the optimized lattice parameters were *a* = 5.17 Å, *b* = 5.16 Å, *c* = 11.76 Å, *α* = *β* = 90⁰, *γ* = 90.15°, which is consistent with previous meta-GGA (*a* = *b* = 5.11 Å, *c* = 11.60 Å, *α* = *β* = *γ* = 90°) and GGA + U calculated results (*a* = *b* = 5.19 Å, *c* = 11.83 Å, *α* = *β* = *γ* = 90°) [37,38], and the lattice constants changed slightly by less than 1% compared with experimental values (*a* = *b* = 5.15 Å, *c* = 11.72 Å, *α* = *β* = *γ* = 90°) [37]. Considering that some of previous studies rotated the BiVO_4_ 90° around the axis *a* and set *b* as the longest axis, *b* was chosen as the longest axis in this calculation, which is consistent with the axes used in other experiments [29,30]. In this case, the lattice parameters were defined as *a* = 5.17 Å, *b* = 11.76 Å, *c* = 5.16 Å, *α* = *γ* = 90°, *β* = 90.15°. After obtaining the optimized unit cell, the *β* was set to 90° and the orthorhombic cell was adopted here. This adjustment does not affect the results and conclusions due to its small error (less than 1%). The side view and top view of a BiVO_4_ unit cell are shown in Figure S1. In order to support the reliability and rationality of te results, the band structure of bulk BiVO_4_ was calculated, as shown in Figure S2. The calculated CBM and VBM related to the Fermi level are 1.72, −0.43 eV, respectively, and the band gap is 2.16 eV for bulk BiVO_4_, which is consistent with previous calculated results (2.17 eV) [39]. In experiments for OER, the improved-photocatalytic-performance high-index facets are mainly on 24-faceted BiVO_4_ and 30-faceted BiVO_4_, and the 24-faceted BiVO_4_ are mainly surrounded by (012), (210), (115) and (511) facets. As for 30-faceted BiVO_4_, (010), (121), (132) and (231) facets cover most of the area of BiVO_4_. The low-index (010) facet has already been investigated in our previous works [40]. Hence, only (012), (210), (115), (511), (121), (132) and (231) are investigated here. In some previous studies, *c* is selected as the longest axis. In this case, the high-index (012), (210), (115), (511), (121), (132) and (231) facets would be defined as (021), (201), (151), (511), (112), (123) and (213), respectively. The (012), (210), (115), (511), (121), (132) and (231) systems are obtained by cleaving the unit cell of bulk BiVO_4_. The thickness of BiVO_4_ in each surface system is larger than 10 Å. The side and top views of optimized structures are shown in Figure 1 and Figure S3, respectively, and the detailed information about thickness and the number of each atom for these structures is shown in Table S1. The thickness is defined by the vertical distance between the highest and lowest atoms for each structure. The (210), (511) and (231) facet can be obtained through a symmetric rotation of (012), (115) and (132) around the longest axis, respectively. Hence, the morphology of (210) is similar to that of (012), (511) is similar to (115), and (231) is similar to (132). Moreover, due to the lattice parameters *a* and *c* of the optimized unit cell BiVO_4_ not being exactly equal, the (012)/(115)/(132) facets and the (210)/(511)/(231) facets are not entirely consistent. Hence, the (012)/(115)/(132) facets probably have negligibly different properties compared with the (210)/(511)/(231) facets.

### 3.2. Electronic Structures

**Figure 2:**
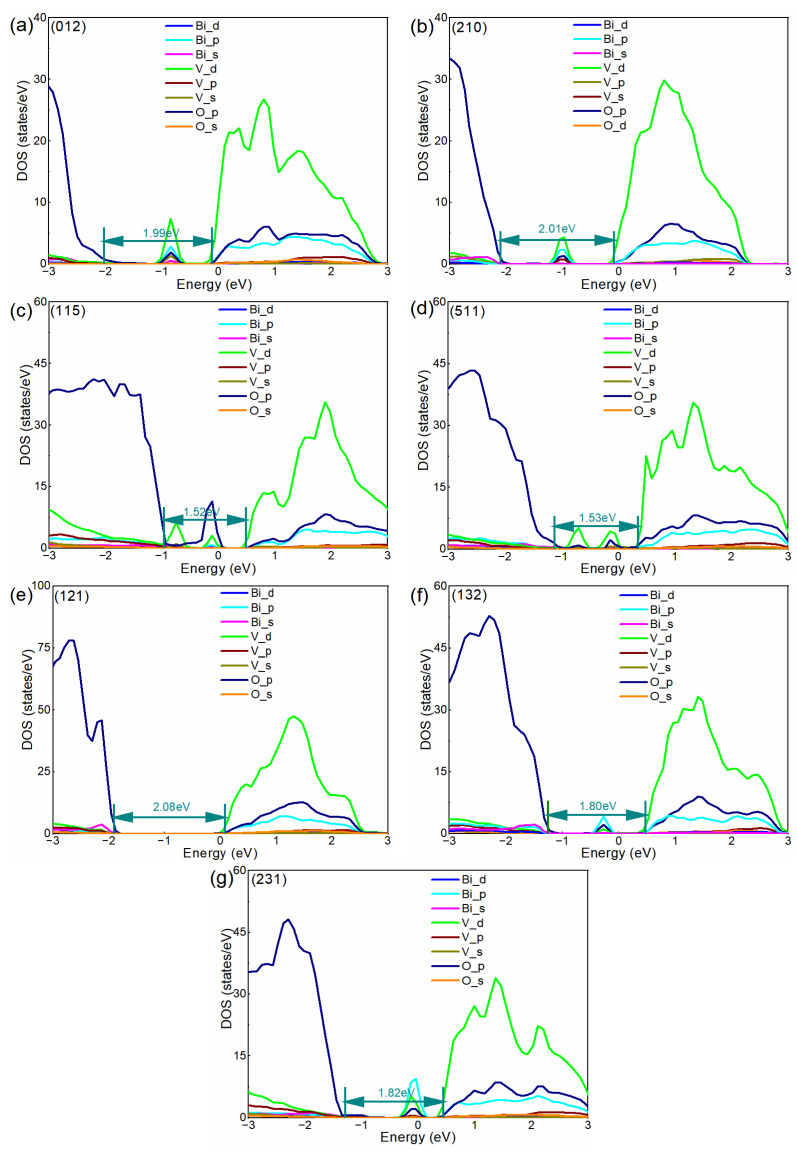
(**a**−**g**) Calculated DOS of BiVO_4_ with different facets. The Fermi level is set to zero.

After obtaining the most stable structures of surface systems, the electronic structure of BiVO_4_ (012), (210), (115), (511), (121), (132) and (231) is investigated by calculating the partial DOS. Here, several layers of atoms for each structure are projected and the calculated results are shown in Figure 2a–g. Clearly, the VBM is mainly composed of O 2p and the CBM is mainly contributed to by V 3d for all of the calculated systems, and the facets do not affect the composition of CBM and VBM significantly. All of the high-index facets show an *n*-type behavior, where the Fermi level is closer to the CBM than VBM. Notably, for (012), (210), (132) and (231), there is one peak that appears in the middle of the band gap, and it can be observed that all the peaks are composed of O 2p and V 3d. The peak of (132)/(231) is very close to the Fermi level, indicating the states might have roughly the same ability to trap electrons and holes. Therefore, the states of (132)/(231) could act as the recombination center, which is not beneficial to improve the PEC performance to some extent. The peak of (012)/(210) is far below the Fermi level, which might have a different ability to trap electrons and holes. In this case, the states could reduce the recombination of electron–hole pairs, which is good for photocatalysis. For (115) or (511), there are two peaks between CBM and VBM. One peak is close to the Fermi level, and this peak is mainly populated by O 2p and V 3d. The other peak is far below the Fermi level, which is mainly contributed to by V 3d. Therefore, the two peaks might have the opposite effect on photocatalysis, and it is hard to establish the combined effect of the two peaks. For (121), there is no peak between CBM and VBM. In our calculation, the high-index facets represent an inclined plane rather than a standard plane, and thus the periodic boundary condition causes the similar morphology to take on different structures. Furthermore, the unit cell of BiVO_4_ is an orthorhombic cell with a slight deformation. These factors probably cause the high-index facets with similar morphology to display slightly different electronic structures. However, these differences do not qualitatively change the electronic structure for the facets with a similar morphology. In our calculation, the electronic structure of (210) is similar to that of (012), (511) is similar to (115), and (231) is similar to (132). Moreover, it can be seen that the band gap and band edge can be modulated by facets. This phenomenon indicates that the oxidation and reduction capacity can probably be modulated according to crystal facet engineering of BiVO_4_.

### 3.3. Band Alignments

**Figure 3:**
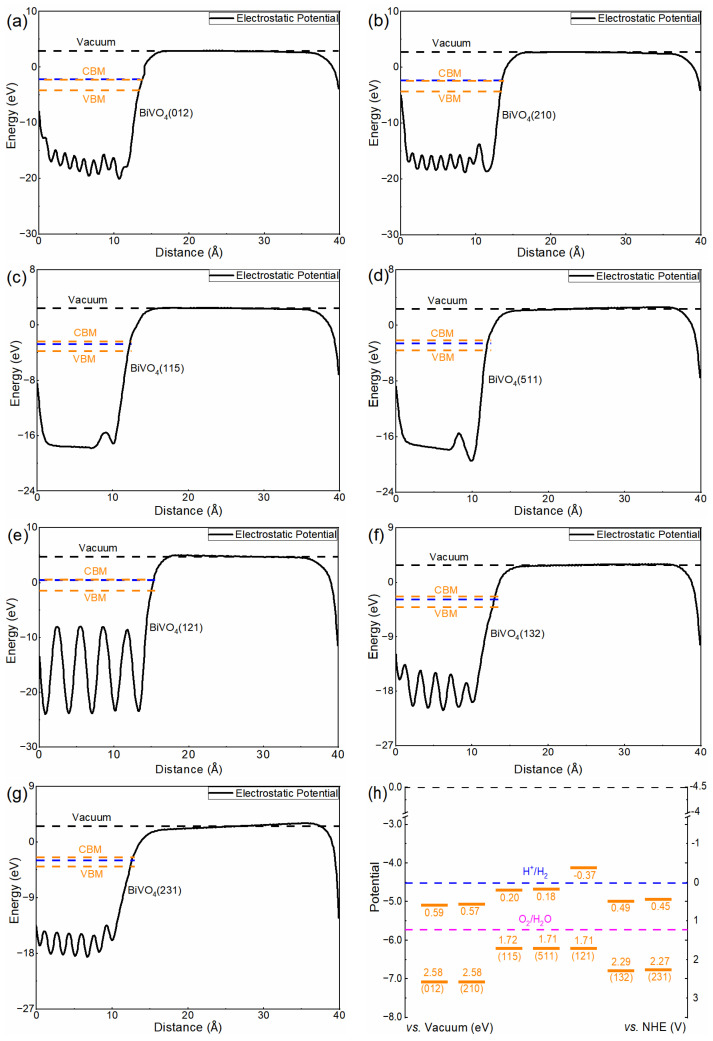
(**a**–**g**) The relative positions of the electrostatic potential of BiVO_4_ with different facets. The blue dashed line represents the Fermi level. (**h**) The band edge potentials related to H^+^/H_2_ and O_2_/H_2_O potentials for BiVO_4_ with different facets. The upper and bottom orange lines represent CBM and VBM, respectively.

The band edge position could determine the oxidation capacity of holes and the reduction of electrons to a great extent, which plays an important role in photocatalytic applications. In this calculation, the band edge position is investigated based on the macroscopic averaging method [41]. The calculated results for different facets are shown in Figure 3a–g. It can be seen that the facets have a great effect on CBM/VBM position. Here, we focus on the distance between CBM/VBM position and vacuum level, and the CBM/VBM position (vs. Vacuum) for each structure is displayed in Table S2. The band edge positions could determine the reduction capacity of photogenerated electrons and the oxidation capacity of photogenerated holes to a great extent. Compared with the band edge positions, it can be inferred that (012) and (210) demonstrate strong oxidation capacity while (121) has a strong reduction capacity. Moreover, the band edge position of (210) is similar to that of (012), (511) is similar to (115), and (231) is similar to (132) in our calculation.

For water splitting, the CBM and VBM should be compared with the reduction potential of H^+^/H_2_ and the oxidation potential of O_2_/H_2_O, respectively. Based on the relationship between the absolute vacuum level and the normal hydrogen electrode (NHE), the CBM and VBM of BiVO_4_ with different facets related to H^+^/H_2_ and O_2_/H_2_O potentials are calculated, as displayed in Figure 3h. It can be seen that the CBM of BiVO_4_ (121) is above the H^+^/H_2_ level, meaning that it has sufficient ability to reduce protons. Meanwhile, the other facets do not have enough reduction ability, and thus the biased voltage is necessary for (012), (210), (115), (511), (132) and (231) to produce H_2_. The VBMs of all of the facets in our calculated systems are below the O_2_/H_2_O potential, indicating they have sufficient oxidation capacity to produce O_2_ without biased voltage.

### 3.4. Stability

Resistance against photocorrsion is important for photocatalysis. An excellent photocatalyst should remain stable for a long time under light illumination. In order to investigate whether the facets have the ability to resist photocorrosion, the stability of BiVO_4_ with different water splitting capacities under light illumination is analyzed. Generally speaking, whether the photocatalyst will be easily corroded is largely determined by the reduction potential ($\varphi_{re}$) and oxidation potential ($\varphi_{ox}$). For water splitting, when the $\varphi_{re}$ of the photocathode can cause a reduction reaction to occur and is higher than the H^+^/H_2_ potential, the photocatalyst will be corroded because of reduction. When the $\varphi_{ox}$ of photoanode can cause an oxidation reaction to occur and is smaller than the O_2_/H_2_O potential, the photocatalyst will be corroded because of oxidation [42]. In the reaction for water splitting, the photogenerated electrons participate in self-reduction while the photogenerated hole takes part in self-oxidization. The possible path for self-reduction can be described as follows [43,44]:

(1) $$2{BiVO}_{4}(s)+6H^{+}+6e^{-}↔2Bi(s)+V_{2}O_{5}(s)+3H_{2}O$$

(2) $$2{BiVO}_{4}(s)+2H^{+}+2e^{-}↔{Bi}_{2}O_{3}(s)+2VO_{2}(s)+H_{2}O$$

(3) $$2{BiVO}_{4}(s)+10H^{+}+10e^{-}↔{Bi}_{2}O_{3}(s)+2V(s)+5H_{2}O$$

(4) $${BiVO}_{4}(s)+8H^{+}+8e^{-}↔Bi(s)+V(s)+4H_{2}O$$

As for self-oxidization, the possible path can be summarized as follows [43,44]:

(5) $$4{BiVO}_{4}(s)+12h^{+}↔4{Bi}^{3+}+2V_{2}O_{5}(s)+3O_{2}$$

(6) $$2{BiVO}_{4}(s)+12h^{+}↔2{Bi}^{3+}+2VO^{3+}+3O_{2}$$

(7) $${BiVO}_{4}(s)+H_{2}O+2h^{+}↔BiVO_{5}+2H^{+}$$

(8) $$4{BiVO}_{4}(s)+H_{2}O+2h^{+}↔{Bi}_{4}O_{7}(s)+2V_{2}O_{5}(s)+2H^{+}$$

Under strongly oxidative conditions, such as largely surface-accumulated holes or an applied high bias potential, there are another two possible paths [43,44]:

(9) $$2{BiVO}_{4}+O_{2}↔2BiVO_{5}$$

(10) $$8{BiVO}_{4}+O_{2}↔2{Bi}_{4}O_{7}+4V_{2}O_{5}$$

In the calculation, the $\varphi_{re}$ and $\varphi_{ox}$ relative to NHE can be determined [42]:

(11) $$\varphi_{re}-\varphi (H^{+}/H_{2})=-[G_{product}-G_{reactans}]/neF$$

(12) $$\varphi_{ox}-\varphi (H^{+}/H_{2})=-[G_{product}-G_{reactans}]/neF$$

where $\varphi (H^{+}/H_{2})$ is the NHE potential, and this value is 0 when the pH is 0. $G_{product}$ and $G_{reactans}$ indicate the Gibbs free energy of products and reactants, respectively. Many of these can be obtained according to the handbook [45]. *n* represents the number of holes or electrons involved in the reduction or oxidization reaction. *e* is the elemental charge, and *F* is the Faraday constant. Moreover, it has been confirmed that the free energy for a compound in the proposed reaction can be approximated via its standard formation enthalpy (Δ*H*) in the calculation [42,46], and the Δ*H* can be defined as follows:

(13) $$\Delta H=E_{tot}-\sum_{i}n_{i}E_{i}$$

where $E_{tot}$ represents the total energy of compound, $n_{i}$ is the number of species *i* atoms that the compound contains, and $E_{i}$ represents the energy of pure element *i* in its conventional reference phases.

Here, some crystal cells are moderately adjusted to enable the structures to satisfy the conditions for calculating Δ*H*, and the structures for calculating Δ*H* are plotted in Figure S4. Detailed information about formation enthalpy can be found in Table S3. It can be seen the (012) and (210) facets have similar Δ*H* in our calculation. Also, the Δ*H* of (115) is similar to that of (511), while (132) is similar to (231). Hence, the morphology, electronic structures, band edge positions and formation enthalpy of the (012), (115) and (132) surfaces are similar to the (210), (511) and (231) surfaces, respectively, and the former could be equivalent to the latter. It can be inferred the results obtained for the (210), (511) and (231) surfaces could be extrapolated for the (012), (115) and (132) surfaces, respectively. Therefore, the $\varphi_{re}$/$\varphi_{ox}$ of (012), (115) and (132) are not calculated here.

**Figure 4:**
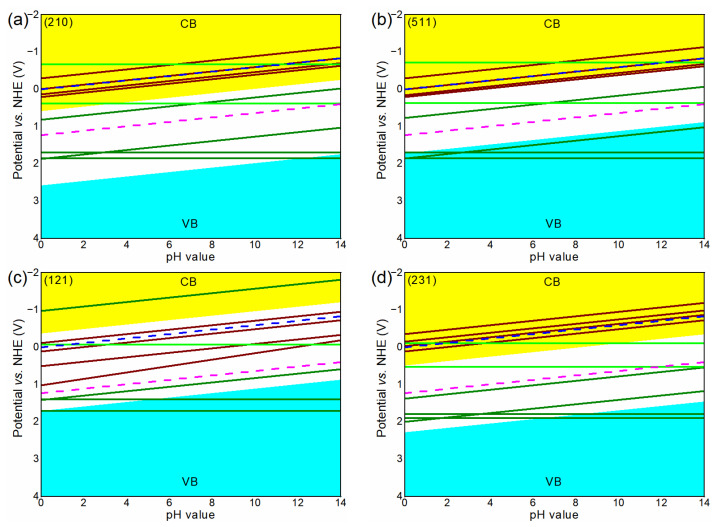
(**a**–**d**) The reduction and oxidation potentials of BiVO_4_ with different surfaces. The wine lines stand for reactions. The green and olive lines represent oxidation reactions with and without strongly oxidative conditions, respectively. The blue and magenta dashed lines are the H^+^/H_2_ and O_2_/H_2_O potential, respectively. The yellow and cyan areas represent the conduction band (CB) and the valence band (VB), respectively.

The results of $\varphi_{re}$ and $\varphi_{ox}$ are shown in Figure 4a–d and Table S4. For the reduction reaction, (210), (511), (121), and (231) have a higher $\varphi_{re}$ than H^+^/H_2_ potential, and thus they would be corroded under light illumination. Therefore, they are not suitable for the production of H_2_ as photocathodes. For the oxidation reaction without strongly oxidative conditions, (210), (511) and (121) have a smaller $\varphi_{ox}$ than O_2_/H_2_O potential, indicating that these facets are easily corroded, while (231) is stable when the strongly oxidative conditions do not exist. Under strongly oxidative conditions, all of the facets in our calculation are unstable and would be oxidized, and this is the reason why the dissolution of BiVO_4_ is largely promoted by illumination as well as high bias potential [47,48]. Generally speaking, a high applied bias potential can increase photocatalytic performance due to it enhancing electron hole separation. However, the high applied bias potential probably induces strongly oxidative conditions, which make the BiVO_4_ photoanode unstable and not good for photocatalysis. The competitive reactions of self-oxidation and water oxidation play an important role for the stability of (231) under high applied potentials.

### 3.5. Overpotential

In order to further analyze the photocatalytic activity, the OER performance of BiVO_4_ (210), (511), (121) and (231) is analyzed according to overpotential based on the CHE model [35]. For OER, there are four steps, and each step contains one electron transfer. The reaction of the OER path can be written as:

(14) $$H_{2}O\left(l\right)+*⇌OH^{*}+H^{+}+e^{-}$$

(15) $$OH^{*}⇌O^{*}+H^{+}+e^{-}$$

(16) $$O^{*}+H_{2}O\left(l\right)⇌OOH^{*}+H^{+}+e^{-}$$

(17) $$OOH^{*}⇌*+O_{2}(g)+H^{+}+e^{-}$$

where * refers to the active site of BiVO_4_, and $OH^{*}$, $O^{*}$ and $OOH^{*}$ represent the adsorbed intermediates in the OER process. Theoretical overpotential (*η*) is related to the largest Gibbs free energy change (Δ*G*) among the four steps:

(18) $$\eta =-max[\Delta G_{{OH}^{*}},(\Delta G_{O^{*}}-\Delta G_{{OH}^{*}}),(\Delta G_{{OOH}^{*}}-\Delta G_{O^{*}}),(4.92-\Delta G_{{OOH}^{*}})]/e-1.23$$

and the Δ*G* is the difference in Gibbs free energy between product and reactant, which can be described as:

(19) $$\Delta G=\Delta E+\Delta E_{ZPE}-T\Delta S$$

in which the Δ*E* is the adsorption energy, and Δ*E*_ZPE_ and Δ*S* represent ZPE and entropy, respectively. *T* is the temperature. At the potential of zero, the relationship of Gibbs free energy meets the conditions:

*G*(H^+^) + *G*(e^−^) = 1/2*G*(H_2_) (20)

*G*(H^+^) + *G*(OH^−^) = *G*(H_2_O) (21)

2*G*(H_2_) + *G*(O_2_) − 2*G*(H_2_O) = 4.92 eV (22)

The free energy of O_2_ is calculated using Equation (22) rather than DFT due to the large error in calculating O_2_ in the VASP program. The structures of adsorbed intermediates, including OH*, O* and OOH*, are shown in Figure S5. Detailed information regarding the calculated total energy, ZPE and entropy is displayed in Table S5.

**Figure 5:**
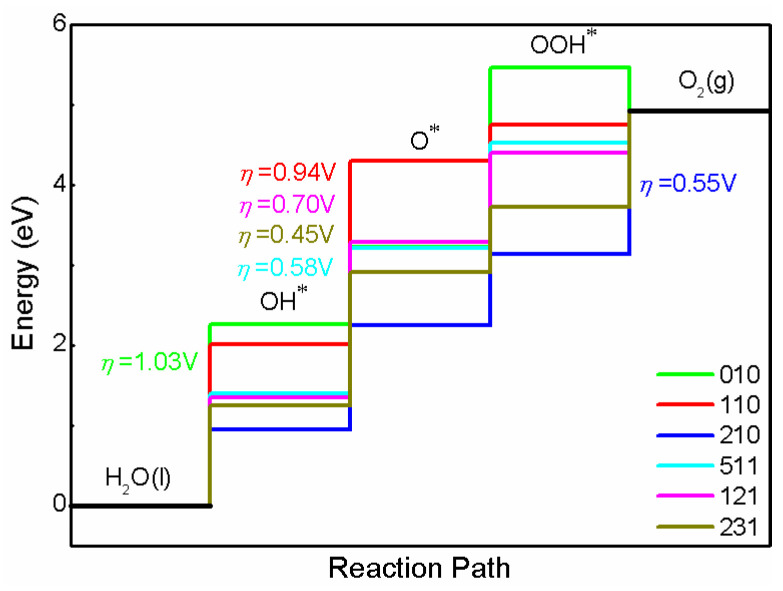
The calculated OER free energy for BiVO_4_ with different facets.

The results for overpotential are plotted in Figure 5. Considering that the actual reaction is taking place in the solution, the implicit solvent model is adopted to correct the free energy. The data for the overpotential of (010)/(110) come from our previous calculation [40], calculated using the same parameter for this work. In order to highlight the effect of solvent correction. The data without solvent correction are also calculated, and the results are plotted in Figure S6. Clearly, the facets impact free energy significantly, whether solvent correction is added or not. Total speaking, the solvent correction has a greater effect on the Gibbs free energy change of OH* and OOH* compared with O*. With the solvent correction, the overpotentials needed for (210), (511), (121) and (231) are 0.55, 0.58, 0.70 and 0.45 V, respectively. When the solvent correction is not considered, the overpotentials are 0.65, 0.62, 0.73 and 0.56 V for (210), (511), (121) and (231), respectively. Therefore, the solvation mainly affects the Gibbs free energy of the adsorbed intermediates OH* and OOH* and further changes the overpotential in our calculated structures. According the calculated overpotentials, the photocatalytic performance trend of these facets is predicted to be (231) > (210) ≈ (511) > (121) > (110) > (010). It should be noted that all of the high-index facets show a lower overpotential than the conventional low-index facets (010) and (110). Although the overpotentials are different for each structure with and without solvent correction, the conclusions do not change. Therefore, these high-index facets might exhibit a better photocatalytic performance than low-index facets (010) and (110) due to their lower overpotential. However, due to (210), (511) and (121) being easily corroded because of self-oxidation in the OER process, only (231) is suitable for OER. In our calculation, (012), (115) and (132) could thus be equivalent to (210), (511) and (121), respectively. It can be inferred that (132)/(231) is the potential surface for OER. The (012)/(210), (115)/(511) and (121) facets have better photocatalytic activity than (010) and (110), but they are easily be corroded. Hence, they should be modulated appropriately in order to resist photocorrosion.

## 4. Conclusions

In summary, we carried out a comprehensive theoretical analysis on the roles of crystal facets in OER using the BiVO_4_. It is found that facets could modulate the electronic structures, band edge positions, stability and overpotential of BiVO_4_ significantly. All of the high-index facets in our calculation show an n-type behavior. The (132)/(231) facet creates one peak near the middle of the band gap, while (012)/(210) creates one peak far below the Fermi level. For (115)/(511), there are two peaks between the CBM and VBM. The band edge position indicates that not all of the facets can produce O_2_ without a biased voltage. The facets could change the overpotential greatly, and the high-index surfaces exhibit a better photocatalytic activity due to their lower overpotential in our calculated systems. However, all of the high-index facets are unstable as a photoanode, except for (132)/(231) when there are no strongly oxidative conditions. Under strong oxidative conditions, all of the facets would be corroded. Generally speaking, (132)/(231) probably has strong oxidation ability, high stability, and low overpotential, making it a potential surface for OER. By controlling the exposed facets, OER performance could be improved to some extent. Our calculation provides important insights into the roles of high-index facets on BiVO_4_ for OER.

## Data Availability

The data that support the findings of this study are available from the corresponding authors upon reasonable request.

## Supplementary Materials

The following supporting information can be downloaded at: https://www.mdpi.com/article/10.3390/nano13132023/s1, Figure S1: The side view and top view of BiVO_4_ unit cell; Figure S2: The band structure of BiVO_4_ unit cell; Figure S3: Top view of geometric structures for BiVO_4_ with different facets; Figure S4: The structures for calculating Δ*H*; Figure S5: The structures of adsorbed intermediate state OH*, O* and OOH* for BiVO_4_ with different facets. The purple, silver, red and white spheres represent Bi, V, O and H, respectively; Figure S6: The calculated OER free energy without solvent correction for BiVO_4_ with different facets; Table S1: The detailed information for BiVO_4_ with different facets. The thickness is defined by the vertical distance between the highest and lowest atoms for each structure; Table S2: The CBM and VBM edge positions of BiVO_4_ with different facets (vs. Vacuum); Table S3: Detailed information about formation enthalpy; Table S4: Calculated reduction and oxidation potentials for different reactions; Table S5: Total energy, zero-point energy and entropy of intermediate states OH*, O* and OOH* for BiVO_4_ with different facets. (T = 300 K).
