# Supplementary material for: Electronic Characteristics, Stability and Water Oxidation Selectivity of High-Index BiVO4 Facets for Photocatalytic Application: A First Principle Study
Source: Nanomaterials (Basel). 2023 Jul 7;13(13):2023. doi: 10.3390/nano13132023 (PMC10343280; doi:10.3390/nano13132023)
Supplement: Supplementary file 1 [file nanomaterials-13-02023-s001.zip › nanomaterials-2478331-supplementary.pdf]

Supplementary Materials

Electronic Characteristics, Stability and Water Oxidation Selectivity of High-Index BiVO4 Facets for Photocatalytic Application: A First Principle Study

Zhiyuan Zhang [1,2], Yuqi Xiang [1,2] and Zhihong Zhu [1,2,*]

[1] College of Advanced Interdisciplinary Studies & Hunan Provincial Key Laboratory of Novel Nano Optoelectronic Information Materials and Devices, National University of Defense Technology, 410073 Changsha, China

[2] Nanhu Laser Laboratory, National University of Defense Technology, Changsha 410073, China

* Correspondence: zzhwcx@163.com

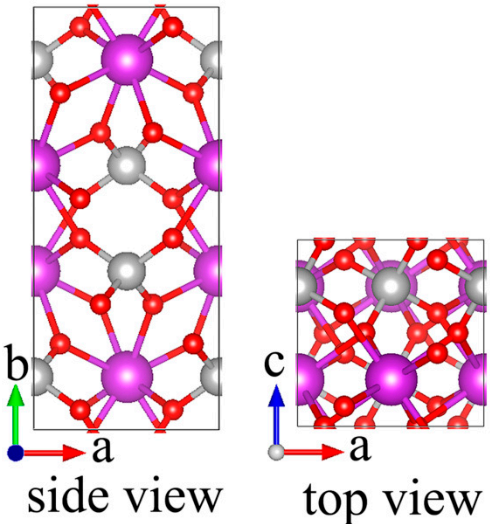

Figure S1. The side view and top view of $BiVO_4$ unit cell.

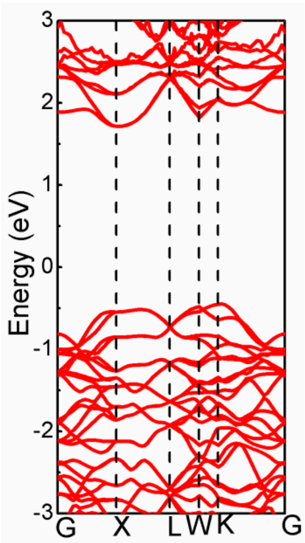

Figure S2. The band structure of $BiVO_4$ unit cell.

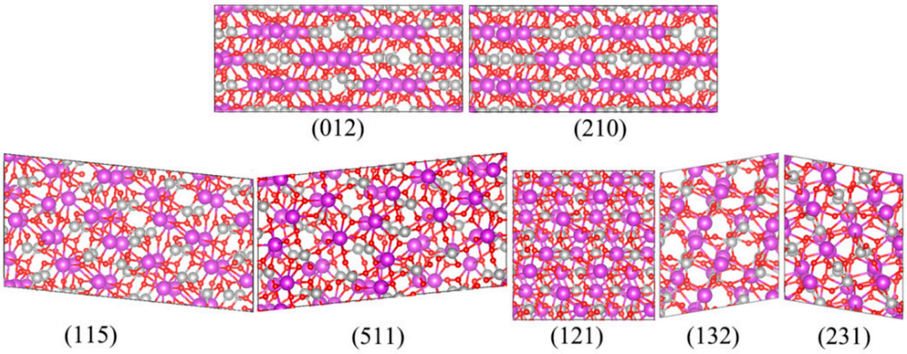

Figure S3. Top view of geometric structures for $BiVO_4$ with different facets.

Facets	Thickness	Lattice parameter			Number of atoms		
		a	*b*	*γ*	Bi	V	O
(012)	12.87Å	10.33Å	24.16Å	90^0	40	40	160
(210)	12.57Å	10.33Å	24.16Å	90^0	40	40	160
(115)	12.83Å	12.86Å	24.33Å	84.11^0	44	44	176
(511)	12.64Å	12.86Å	24.33Å	95.88^0	44	44	176
(121)	14.80Å	13.86Å	14.61Å	90^0	40	40	156
(132)	13.42Å	11.55Å	13.86Å	99.55^0	28	26	104
(231)	13.30Å	11.55Å	13.66Å	80.41^0	26	28	104

Table S1. The detailed information for $BiVO_4$ with different facets. The thickness is defined by the vertical distance between the highest and lowest atoms for each structure.

Facets	CBM (eV)	VBM (eV)
(012)	-5.09	-7.08
(210)	-5.07	-7.08
(115)	-4.70	-6.22
(511)	-4.68	-6.21
(121)	-4.13	-6.21
(132)	-4.99	-6.79
(231)	-4.95	-6.77

Table S2. The CBM and VBM edge positions of $BiVO_4$ with different facets (*vs*. Vacuum).

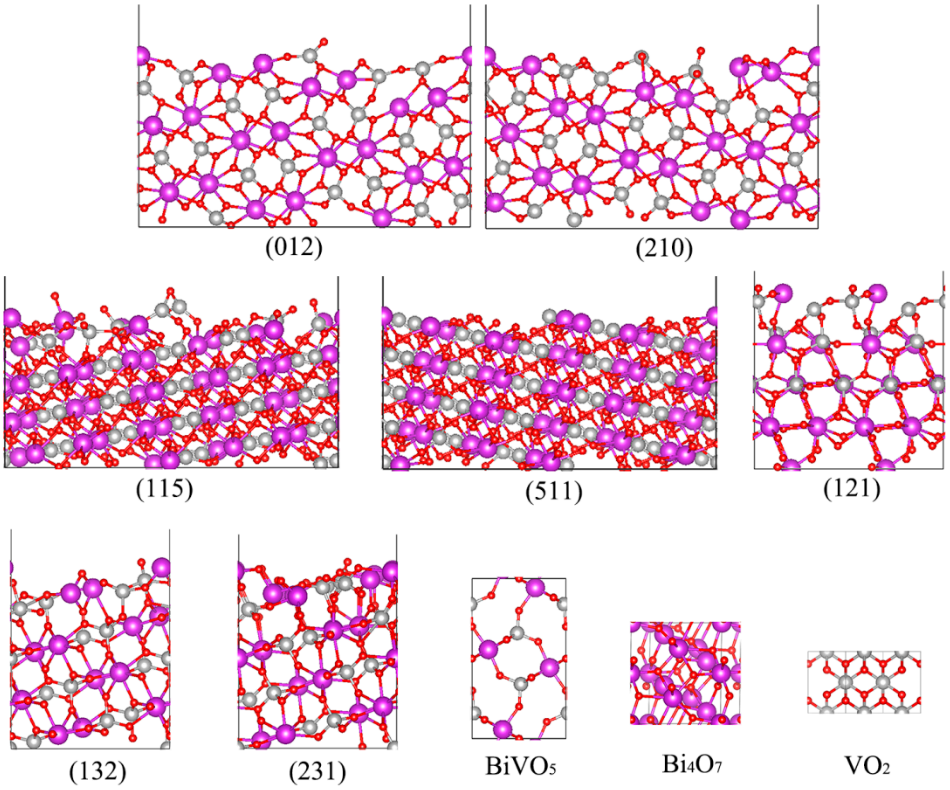

Figure S4. The structures for calculating Δ*H*.

Structure	Δ*H*	Ref.
$BiVO_4$ (012)	-11.877	Calculated
$BiVO_4$ (210)	-11.869	Calculated
$BiVO_4$ (115)	-11.852	Calculated
$BiVO_4$ (511)	-11.863	Calculated
$BiVO_4$ (121)	-10.983	Calculated
$BiVO_4$ (132)	-12.158	Calculated
$BiVO_4$ (231)	-12.163	Calculated
V_2O_5	-16.071	Handbook
Bi_2O_3	-5.948	Handbook
VO_2	-7.551	Calculated
Bi^{3+}	1.270	Handbook
VO^{3+}	-2.010	Handbook
$BiVO_5$	-11.109	Calculated
Bi_4O_7	-16.703	Calculated
H_2O	-2.962	Handbook

Table S3. Detailed information about formation enthalpy.

	(012)	(115)	(121)	(132)
R1	0.201	0.209	0.499	0.107
R2	0.129	0.154	1.023	-0.152
R3	-0.299	-0.295	-0.121	-0.356
R4	-0.004	-0.001	0.108	-0.039
R5	1.704	1.696	1.406	1.798
R6	1.857	1.852	1.707	1.902
R7	1.865	1.853	1.418	2.003
R8	0.813	0.763	-0.976	1.375
R9	0.384	0.372	-0.063	0.525
R10	-0.667	-0.719	-2.456	-0.107

Table S4. Calculated reduction and oxidation potentials for different reactions.

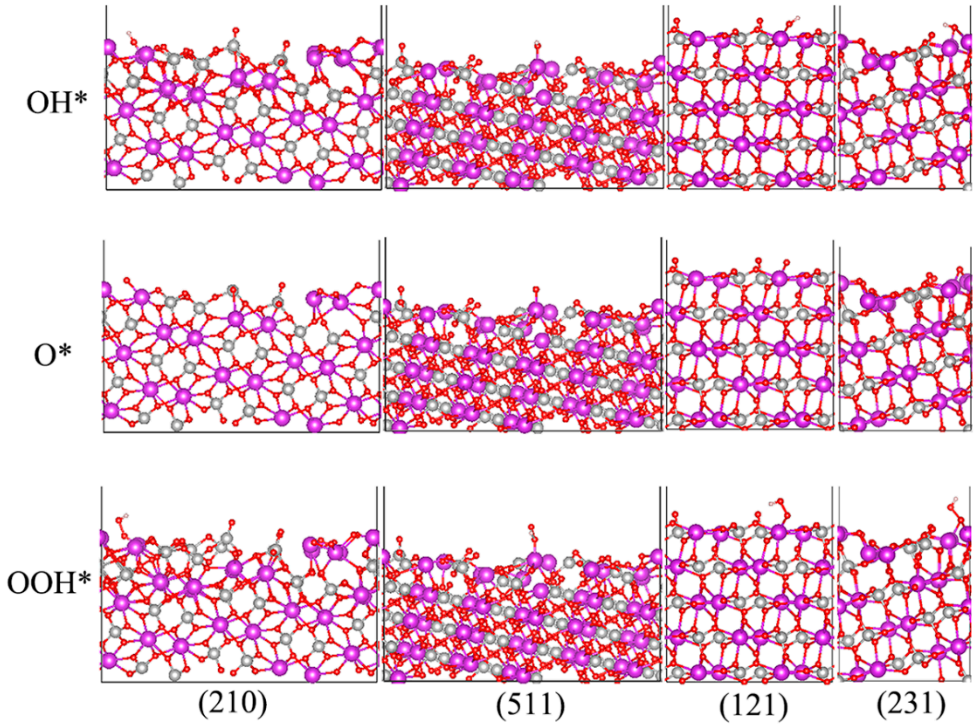

Figure S5. The structures of adsorbed intermediate state OH*, O* and OOH* for $BiVO_4$ with different facets. The purple, silver, red and white spheres represent Bi, V, O and H, respectively.

Facets	E(eV)	E_{slab}(eV)	ΔE_{ZPE}	ΔS(eV)	ΔE_{ZPE} - TΔS(eV)
OH*					
(012)	-1717.056	-1706.922	0.342	0.000417	0.267
(115)	-1912.077	-1902.376	0.352	0.000433	0.281
(121)	-1731.059	-1721.304	0.336	0.000355	0.288
(132)	-1133.615	-1123.791	0.323	0.000404	0.265
O*					
(012)	-1712.099	-1706.922	0.046	0.000315	0.010
(115)	-1906.591	-1902.376	0.051	0.000271	0.008
(121)	-1725.445	-1721.304	0.042	0.000291	-0.001
(132)	-1128.299	-1123.791	0.027	0.000365	-0.018
OOH*					
(012)	-1722.303	-1706.922	0.397	0.000550	0.287
(115)	-1916.388	-1902.376	0.413	0.000608	0.298
(121)	-1735.465	-1721.304	0.423	0.000620	0.329
(132)	-1138.547	-1123.791	0.364	0.000823	0.240

Table S5. Total energy, zero-point energy and entropy of intermediate states OH*, O* and OOH* for $BiVO_4$ with different facets. (T = 300K).

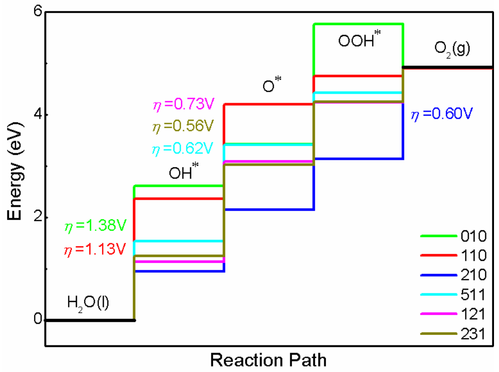

Figure S6. The calculated OER free energy without solvent correction for $BiVO_4$ with different facets.
